# Enzymatic Carboxylation of Resorcinol in Aqueous Triethanolamine at Elevated CO_2_ Pressure

**DOI:** 10.3390/molecules29010025

**Published:** 2023-12-19

**Authors:** Daniel Ohde, Benjamin Thomas, Paul Bubenheim, Andreas Liese

**Affiliations:** Institute of Technical Biocatalysis, Hamburg University of Technology, 21073 Hamburg, Germany; bennie@gmx.de (B.T.); paul.bubenheim@tuhh.de (P.B.)

**Keywords:** CO_2_ fixation, carboxylation, tetrabutylammonium, elevated pressure, biocatalysis

## Abstract

The fixation of CO_2_ by enzymatic carboxylation for production of valuable carboxylic acids is one way to recycle carbon. Unfortunately, this type of reaction is limited by an unfavourable thermodynamic equilibrium. An excess of the C1 substrate is required to increase conversions. Solvents with a high CO_2_ solubility, such as amines, can provide the C1 substrate in excess. Here, we report on the effect of CO_2_ pressures up to 1100 kPa on the enzymatic carboxylation of resorcinol in aqueous triethanolamine. Equilibrium yields correlate to the bicarbonate concentration. However, inhibition is observed at elevated pressure, severely reducing the enzyme activity. The reaction yields were reduced at higher pressures, whereas at ambient pressure, higher yields were achieved. Overall, CO_2_ pressures above 100 kPa have been demonstrated to be counterproductive for improving the biotransformation, as productivity decreases rapidly for only a modest improvement in conversion. It is expected that CO_2_ carbamylation intensifies at elevated CO_2_ pressures, causing the inhibition of the enzyme. To further increase the reaction yield, the in situ product precipitation is tested by the addition of the quaternary ammonium salt tetrabutylammonium bromide.

## 1. Introduction

Benzoic acid decarboxylases catalyse the decarboxylation of benzoic acids to their respective phenols, but they also work in the reverse direction to carboxylate phenols. Their ability to carboxylate various phenols selectively, such as resorcinol, resveratrol, and gnetol [1], via the utilization of CO_2_, attracted attention in the scientific community [2,3,4]. The major challenge of carboxylation reactions are the unfavourable thermodynamics, which prevent reaching high conversions under conventional conditions. CO_2_ has an especially high thermodynamic stability with a standard Gibbs free energy formation of −394 kJ mol^−1^ [5]. Carbon ligases (EC 6) overcome the energy barrier by performing substrate activation with energy-rich, but also expensive, cofactors such as ATP. The (de)carboxylases (EC 4) do not use cofactors and require different approaches to enhance the CO_2_ fixation. To make enzymatic carboxylations viable for industrial production of valuable carboxylated chemicals [4], research should focus on finding strategies to enhance the conversions. For example, the impact of the concentration of the C1 substrate, either by increasing the concentration of supplied bicarbonate salts, or increased CO_2_ partial pressures in aqueous systems, were studied [6]. However, the application of 2 M KHCO_3_ under ambient pressure and a CO_2_ partial pressure of 800 kPa only resulted in a conversion of around 25% for the carboxylation of catechol [6]. Substituting KHCO_3_ with other bicarbonate salts was found to have a strong impact on both the enzyme activity and the conversion. This dependency on bicarbonates was found to correlate to the Hofmeister series, but still, the conversion could not be sufficiently enhanced [7]. Higher conversions were reported when different organic solvents and ionic liquids were supplemented [7]. Using amines as solvents is particularly promising as amines capture gaseous CO_2_ and provide the biotransformation with a high C1 substrate concentration [8]. Recently, we showed that aqueous triethanolamine can be loaded very efficiently with gaseous CO_2_ by using microbubble sparging in a bubble column setup [9]. Moreover, we discovered that the equilibrium yield depends on the HCO_3_^−^ species, which was the main species of the dissolved inorganic carbon (DIC) content [10]. Similarly, Fan et al. have recently shown that the carboxylation of catechol by a 2,3-dihydroxybenzoic acid decarboxylase from *Aspergillus oryzae* depends on the aqueous CO_2_ concentration, with a high concentration of HCO_3_^−^ being required under alkaline conditions [11].

In contrast to approaches that increase the substrate concentration, the removal of the formed product can also increase the conversion. In the literature, several adsorber materials have been used [12,13]. However, none of these adsorbers are selective enough to remove the product without binding large amounts of substrate, too [12,13]. Another approach used enzyme cascades making use of the product from the carboxylation in subsequent reactions. This approach has been shown to be compatible for the production of amino acids [14]. However, this cascade needs to be optimized as the product titer achieved was very low, and the process is generally more expensive than traditional processes. Enzyme development and production costs were the main contributors to the overall process cost. A major improvement was achieved by Ren et al., who found that certain quaternary ammonium salts can selectively initiate crystallization of some benzoic acid derivatives, accomplishing an efficient in situ product removal enabling near-full conversions [15,16]. The main disadvantages of this technique are that a suitable pair of quaternary ammonium ions and benzoic acid derivatives must be found and that a large amount of bicarbonate salt is wasted. Dealing with unreacted bicarbonate salt is a concern that all such systems have in common. However, this can be mitigated by recycling the unused bicarbonate salt or even circumvented completely by using gaseous CO_2_ in combination with a reaction medium that has a high CO_2_ solubility. A promising system utilizes amines used for carbon capture in the industry to directly provide the C1 substrate for the biotransformation [10]. There, much higher conversions are already possible, and the combination with the previously mentioned quaternary ammonium precipitation method at low amine concentrations further increases conversions [10].

**Figure 1:**
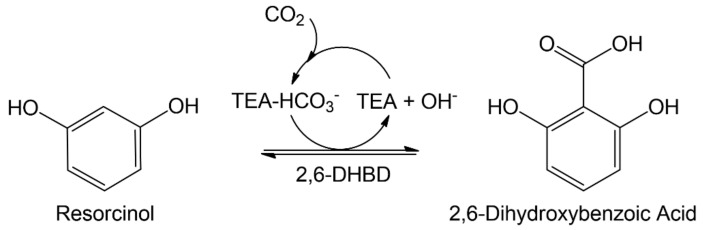
Enzymatic carboxylation of resorcinol to 2,6-dihydroxybenzoic acid (2,6-DHBA) by 2,6-DHBA decarboxylase (2,6-DHBD) in aqueous triethanolamine (TEA) saturated with CO_2_.

Here, we report on the combination of the aqueous amine system with increased CO_2_ pressure and quaternary ammonium precipitation and its effect on the investigated enzymatic carboxylation. As the model reaction system, the carboxylation of resorcinol by 2,6-dihydroxybenzoic acid (2,6-DHBA) decarboxylase (2,6-DHBD) from *Rhizobium* sp. in aqueous triethanolamine is investigated (Figure 1). An, up to now, unrecognized and critical inhibition is shown to limit the biotransformation at elevated CO_2_ pressures.

## 2. Results and Discussion

### 2.1. Carboxylation at Elevated CO_2_ Pressure

A usual strategy to increase conversion of the thermodynamic limited carboxylation is to perform the reaction under excess C1 substrate conditions. Compared to the conventional approach where the C1 substrate is supplied by addition of bicarbonate salts, the amine-mediated system utilizes gaseous CO_2_ to provide the C1 substrate. Bicarbonate salts are largely limited by their solubility in the particular medium, whereas the dissolved inorganic carbon (DIC) concentration is primarily dependent on the CO_2_ pressure.

**Figure 2:**
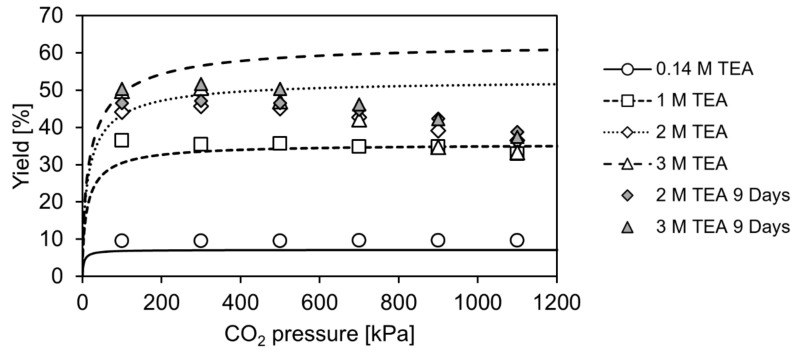
Theoretical equilibrium yield (lines) and experimentally determined reaction yield (symbols) for the enzymatic carboxylation of 80 mM resorcinol to 2,6-dihydroxybenzoic acid up to 1200 kPa CO_2_ in 0.14 M to 3 M aqueous triethanolamine. Samples were incubated for 2 days and 9 days. Only the 9-day samples of 2 and 3 M aqueous TEA are shown as their yield still increased.

In this work, the carboxylation of resorcinol is investigated at pressures up to 1100 kPa of pure CO_2_ to determine the influence of the pressure on the reaction yield. For calculation of CO_2_ solubility in aqueous TEA, the correlation of Jou et al. (1985) is utilized [17]. Additionally, the pH of the reaction solution can be calculated with this correlation as it incorporates the method of Kent and Eisenberg (1976) using equilibrium constants to correlate solubility data [18]. This approach is applied to calculate theoretical concentrations of CO_2_/H_2_CO_3_, HCO_3_^−^ and CO_3_^2−^ in aqueous TEA for the investigated pressure range. In combination with the equilibrium constant, determined in a previous work [10], theoretical equilibrium yields were calculated and compared with experimental reaction yields, as shown in Figure 2.

At 100 kPa, predicted and experimental equilibrium yields fit well. In our previous work [10], lower yields were reported for the investigated aqueous TEA concentrations. For example, at 3 M aqueous TEA, an equilibrium yield of around 40% was reported, whereas around 50% are achieved in this work. However, these values cannot be directly compared as a crucial condition is changed. In the previous work, aqueous TEA was only presaturated, and then resorcinol and 2,6-DHBD were added, diluting the CO_2_ saturated TEA. Thereby, these experiments do not correspond to conditions at 100 kPa CO_2_ pressure, which was accounted for in the study. For example, in Figure 5 (right) and Figure 6 of Ohde et al. (2021) [10], it was predicted that reaching 50% equilibrium yield is possible at a dissolved inorganic content DIC of around 1700 mM and 3 M aqueous TEA. A DIC of around 1700 mM can be reached at 3 M aqueous TEA at 100 kPa. In turn, reaching 50% equilibrium yield at 3 M aqueous TEA is confirmed in the current study (see Figure 2). This is achieved by pressurization of the reactor or flushing with CO_2_ in the case of the control reactor after the initial CO_2_ presaturation and start of the reaction. In this way, the intended CO_2_ pressure is reached.

Comparing the predicted and the experimental yields for the whole pressure range tested in this study, it is observed that the yield prediction fits the experimental data only for the lower aqueous TEA concentrations (approximately up to 1 M). At high TEA concentration, the applied model apparently overestimates the yields, with the difference between prediction and experimentally obtained data increasing at higher applied CO_2_ pressure. No side product was formed that might explain the difference, as conversion and yield were identical in all experiments. Another explanation for the deviation might be that the equilibrium reaction yield was not yet reached in the reaction after 2 days of reaction time. In this case, the reaction yield cannot fit the equilibrium yield, which is predicted by the applied model. Therefore, additional experiments are performed, where the reaction time is extended to check if the reaction yield would still increase. The measurements confirmed that the reaction equilibrium was not achieved after 2 and even 9 days under elevated CO_2_ pressure. A further control experiment was performed. Instead of testing the carboxylation, the decarboxylation of 80 mM 2,6-DHBA up to 1200 kPa was performed. The decarboxylation is favoured in terms of thermodynamics, but even after 10 days, the equilibrium yield was not reached as observed in the previous experiments. The additional feature of this experiment is that resorcinol-dependent deactivation of the enzyme can be neglected due to resorcinol not being present at the start and only accumulating in the course of the reaction.

**Figure 3:**
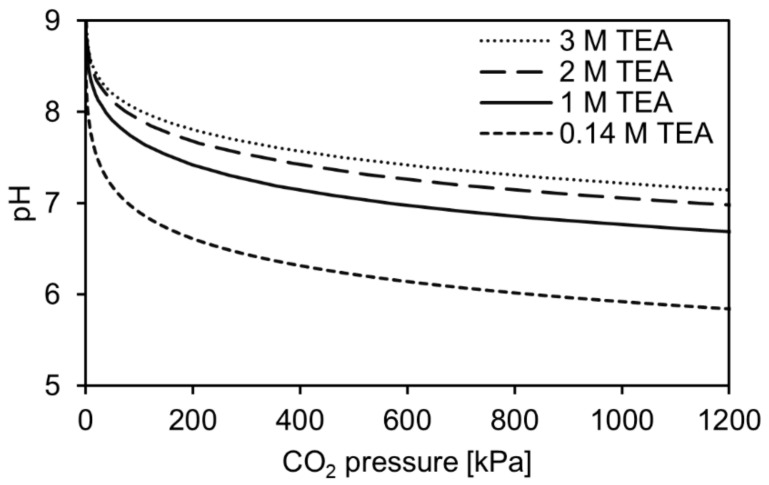
Calculated pH profiles for different concentrations of aqueous triethanolamine (TEA) solutions at 25 °C and CO_2_ pressures up to 1200 kPa. The calculation was performed according to Jou et al. (1985) [17].

Considering a different type of enzyme deactivation, for example, due to a pH shift, it is unlikely that a pH-dependent deactivation has a major negative impact on the enzyme stability and activity in the investigated pressure range. At 100 kPa, the pH was measured to be in the neutral, up to slightly alkaline pH range for higher TEA concentrations. As the experimental setup made it impossible to measure the pH at elevated pressure, the pH values were instead calculated with the applied model as described above. In Figure 3, the pH-pressure profile up to 1200 kPa is shown for the investigated aqueous TEA concentrations. Initially, the pH decreases rapidly until being buffered by the TEA and the bicarbonate equilibrium. In this way, the slightly alkaline pH is lowered to a more neutral pH by loading the aqueous TEA with CO_2_. Only when using 0.14 M TEA, the pH gets slightly acidic, reaching a value around pH 6 above 800 kPa pressure. Instead of having a negative effect, a positive effect is expected as the enzyme is reported to be most active around the neutral pH region [19].

**Figure 4:**
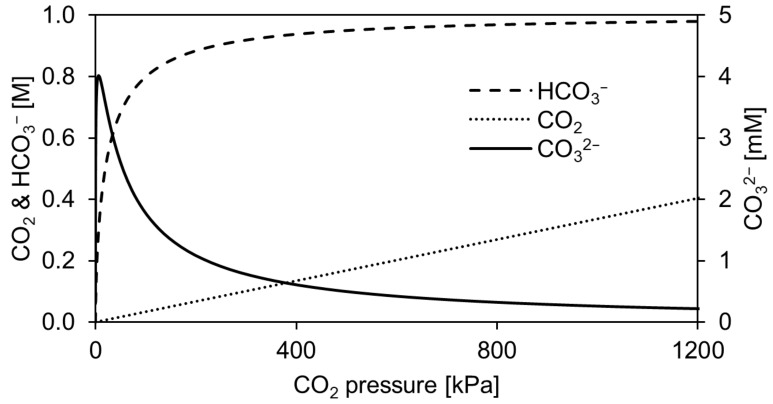
Dissolved concentration of CO_2_/H_2_CO_3_, HCO_3_^−^, and CO_3_^2−^ in 1 M aqueous triethanolamine up to 1200 kPa at 25 °C.

An additional effect of the lowered pH is that the mole fraction and concentration of CO_2_ increases. In turn, this is expected to increase the enzyme activity as demonstrated in a previously published kinetic investigation [10]. In Figure 4, the concentration profile of the different DIC species is shown for 1 M aqueous TEA. It can be seen that both CO_2_ and HCO_3_^−^ concentrations increase, whereas the CO_3_^2−^ concentration has a maximum around 6 kPa, where the pH is calculated to be 8.5. The increase in CO_2_ and HCO_3_^−^ concentrations cannot readily explain the reduced enzyme activity, as CO_2_ is reported to be the most likely active C1 substrate, whereas HCO_3_^−^ largely determines the equilibrium yield at a CO_2_ pressure of 100 kPa [10]. An inhibition seems to occur at high CO_2_ pressures, which could be related to reversible or irreversible carbamylation of amino acids within the enzyme structure. Prominent examples found in nature are ribulose-1,5-bisphosphate carboxylase/oxygenase and haemoglobin, which are both CO_2_ regulated. In haemoglobin, the N-terminal valine is carbamylated forming a carbamate [20,21] that modulates the affinity to bind oxygen at physiological conditions [22]. In the case of ribulose-1,5-bisphosphate carboxylase/oxygenase, both Mg^2+^ and inorganic carbon act as cofactors [23] enabling the enzyme to fix atmospheric CO_2_. In the underlying mechanism to activate the enzyme, CO_2_ needs to bind to an alternative site on the enzyme to form a carbamate as demonstrated in several studies [24,25,26].

In general, carbamate formation can occur in all proteins at the N-terminal amino acid and amino acids with a free amino group such as lysine [27]. The process is usually reversible, and it was found that CO_2_-mediated posttranslational modification of proteins is relevant even at physiological conditions and impacts protein biochemistry [28]. However, it is known that the three-dimensional structure of enzymes may be significantly changed at higher CO_2_ pressures, which affects their stability and may cause denaturation [29]. Consequently, a loss of their activity occurs. Supercritical CO_2_ treatment for enzyme deactivation is, for example, commonly applied in food processing [30,31]. The inactivation by supercritical CO_2_ could be predominantly attributed to the pH-lowering effect during treatment as stated by Balaban et al. [32]. As previously discussed, it is unlikely that a pH-dependent deactivation occurred due to only slight changes in pH being calculated due to TEA acting as a buffer. Nonetheless, deactivation can even occur at subcritical CO_2_ conditions [33]. However, the chance for minor structural changes is higher, which may induce a reversible alternative active protein state with altered enzyme activity, specificity, and stability [34]. As only conditions up to 1100 kPa were investigated in this work, it remains open if reversible or irreversible changes occurred that might explain the decrease in 2,6-DHBD activity. In a future study, it will be investigated whether the enzyme regains activity after an incubation at high CO_2_ pressure without substrate (resorcinol or 2,6-DHBA).

Overall, this inhibition prevented reaching the equilibrium yield during the investigated reaction time. The presented data demonstrate that performing enzymatic carboxylation under elevated pressure up to 1100 kPa is an unfeasible strategy to enhance equilibrium yields compared to ambient CO_2_ pressure conditions.

### 2.2. Precipitation at Elevated CO_2_ Pressure

**Figure 5:**
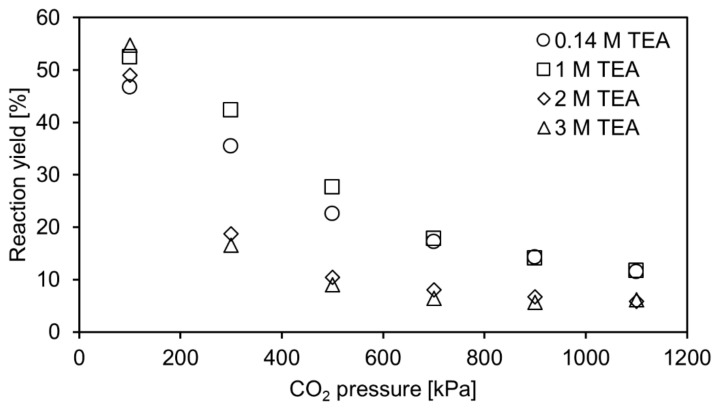
Reaction yield for the enzymatic carboxylation of 80 mM resorcinol to 2,6-dihydroxybenzoic acid with 350 mM tetrabutylammonium bromide under elevated CO_2_ pressure in aqueous triethanolamine (TEA) after 2 days.

Under ambient conditions (30 °C, atmospheric pressure), quaternary ammonium salts such as tetrabutylammonium (TBA) salts are reported to enhance the conversion of the enzymatic carboxylation of hydroxylated benzenes [15,16]. This effect was also reported to work in the aqueous TEA system; however, at greatly reduced efficiency [10]. This approach has now been tested at elevated CO_2_ pressure to see if pressure has a positive effect on the reaction yield. Therefore, the enzymatic carboxylation of resorcinol to 2,6-DHBA was performed with TBA-bromide (TBA-Br) at elevated pressure. In Figure 5, the determined reaction yields are shown. Overall, the addition of TBA-Br improves the reaction yield at 100 kPa CO_2_ pressure, especially for 0.14 M and 1 M TEA, which is consistent with previous data [10]. However, the results demonstrate that the reaction yield decreases at higher pressures with reduced yields. For example, the yields are reduced by only around 12% for 1 M TEA at 1100 kPa instead of by 35% without the addition of TBA-Br, as shown in Figure 5.

The underlying mechanism by which CO_2_ pressure causes a strong effect on the reduction of reaction yield when adding TBA-Br is not fully understood. It is important to note that the mass balance is closed for all experiments and no side product formation is detected. To ensure that the quantification by HPLC is not distorted by formed precipitates, trifluoroacetic acid was added directly to the reaction vessels to stop the reaction and dissolve any formed precipitate. Therefore, side product formation and product contained in any solids can be excluded to cause the yield reduction. A possible reason is that the increased CO_2_ carbamylation causes an enzyme inhibition, which prevents reaching the equilibrium yield as discussed above. It is known that blends of amines with quaternary ammonium salts have a higher CO_2_ solubility [35], which would enhance carbamate formation. This explains part of the observed behaviour. As a consequence of carbamates forming on the enzyme backbone, TBA is expected to enter in electrostatic interactions with the negatively charged carbamate. In turn, the enzyme structure is disrupted, causing a loss in activity. A detailed investigation is required to gain a deeper understanding of the underlying mechanism for the observed behaviour. However, at this stage, it is already clear that it is not feasible to perform the enzymatic carboxylation at elevated pressure when using TBA-Br or most likely any other quaternary ammonium salts.

## 3. Materials and Methods

### 3.1. General

All chemicals, except triethanolamine, were sourced from Sigma-Aldrich (Darmstadt, Germany) with purities of ≥99%. Triethanolamine was purchased from Carl Roth (Karlsruhe, Germany). As for CO_2_, carbon dioxide 4.5 (≥99.995%), purchased from Linde (Pullach, Germany), was utilized.

### 3.2. Production of His-Tagged 2,6-Dihydroxybenzoic Acid Decarboxylase

The original plasmid vector (pET21a+) containing the gene for 2,6-dihydroxybenzoic acid decarboxylase from *Rhizobium* sp. strain MTP-10005 was provided by Prof. Kurt Faber and Dr. Silvia Glueck (Graz University, Graz, Austria). The preparation of the plasmid is described in Wuensch et al. (2013) [7]. For the transformation, *E. coli* BL21(DE3) cells were used. These transformed cells were provided by Dr. Lorenzo Pesci (Hamburg University of Technology, Hamburg, Germany). The detailed transformation procedure is described in Pesci et al. (2015) [6].

Overexpression of 2,6-DHBD was performed in a Luria Bertani [36] medium supplemented with 100 µg/mL of ampicillin in shake flasks according to Pesci et al. (2015) [6]. Cells were harvested by centrifugation at 8000 rpm (11,325 rcf) for 20 min at 4 °C. Cells were washed with 50 mM KPi pH 7.5, and the previously described centrifugation step was repeated. Several of these washing steps were performed, where the supernatant was discarded.

Cell-free extract was prepared by suspending wet cells in 50 mM KPi pH 7.0 to a concentration of 4 g/mL and performing a cell disruption by sonication. The sonication was performed with a 3 mm tip at 70% power using a BANDELIN Sonopuls from BANDELIN electronic (Berlin, Germany). Overall, three cycles were performed, each consisting of three minutes of sonication and a subsequent cooling of the sample on ice for three minutes. After sonication, the sample was centrifuged at 20,000 rpm (48,384 rcf) for 20 min at 4 °C. The obtained supernatant was filtrated using a 0.45 μm filter. Afterwards, the cell-free extract was cooled at 4 °C for subsequent enzyme purification.

For enzyme purification, immobilized metal affinity chromatography in a packed bed column using 90 μm beads of Nickel Sepharose^®^ 6 Fast Flow from Cytiva, Marlborough, MA, USA was performed according to Pesci et al. (2015) [6]. For the final buffer exchange, 50 mM KPi pH 7.0 was used. The purified enzyme was aliquoted and stored at −20 °C. Several batches of purified 2,6-DHBD were produced and combined.

### 3.3. Enzymatic Reactions at Elevated CO_2_ Pressure

In all experiments, purified 2,6-DHBD from one combined batch was used. Reactions were carried out in open 1.5 mL HPLC screw cap vials placed in high-pressure reactors consisting of stainless steel cylinders capped with bronze lids. Several vials can be placed in these reactors, which have a volume of 160 mL and can be operated at pressures of up to 400 bar.

First, reaction mixtures without purified 2,6-DHBD were presaturated with CO_2_ by bubbling through a cannula acting as a sparger until the pH was constant. Then, the enzyme was added to start the reaction. Afterwards, 80 mM of resorcinol and 16.4 U/mg enzyme were used as final concentrations. The enzyme activity was determined according to Ohde et al. (2021) [10]. Immediately after starting the reaction, the vials were placed in the high-pressure reactor, which was flushed three times with CO_2_ to remove air. Then, the reactors were pressurized with CO_2_. Each batch was incubated at room temperature without shaking or stirring.

Control experiments were performed in aluminium reactors. Similar to the high-pressure reactors, these reactors have a volume of 160 mL and were flushed with CO_2_, as well. In this way, the condition for ~100 kPa CO_2_ pressure was ensured.

### 3.4. Enzymatic Reactions at Elevated CO_2_ Pressure with TBA-Br

In situ product precipitation experiments were performed similar to the procedure described in Section 3.3. The only difference was that resorcinol and tetrabutylammonium bromide were added after CO_2_ saturation of aqueous TEA solutions. This was done to prevent precipitates already forming during CO_2_ gassing, as was the case when resorcinol and TBA-Br were added beforehand. The final concentration of resorcinol and TBA-Br in the reactions were 80 mM and 350 mM, respectively. The reaction was started by adding 2,6-DHBD (16.4 U/mg).

### 3.5. Analytics

An RP-HPLC method was used for quantification of resorcinol and 2,6-DHBA. The sample preparation was created by mixing a sample in a ratio of 1:1 with trifluoroacetic acid (TFA). This stopped the reaction and removed amine-bound CO_2_. For samples containing TBA-Br, water and acetonitrile were added in a volume ratio of 1:1:1:1 (sample:TFA:water:ACN). This was done to dissolve formed precipitates. After mixing and complete degassing, which was indicated by no more (CO_2_) bubbles being formed, a centrifugation was performed for 5 min at 13,000 rpm. The supernatant was analysed in an Agilent (Waldbronn, Germany) LC-1100 HPLC system. For separation, a LichroCART 250-4 Lichrospher 100 RP column (5 μm) was used. For analyte detection, the HPLC was equipped with a diode array detector. A gradient method was applied using solutions of acetonitrile with 0.1% (*v*/*v*) TFA and water with 0.1% (*v*/*v*) TFA. The applied gradient is described in a previously reported protocol [37]. Typical retention times were 7.0 min and 8.3 min for resorcinol and 2,6-DHBA, respectively.

## 4. Conclusions

The enzymatic carboxylation of resorcinol to 2,6-dihydroxybenzoic acid can be enhanced by the application of elevated CO_2_ pressure. However, when applying pressures above 100 kPa CO_2_, the increase in conversion is not sufficient to justify the application of higher pressure. This is especially supported by two reasons. First, there would be the need for a more expensive setup that supports biotransformations at elevated pressure. Second, it was found that elevated pressure up to 1100 kPa has a counterproductive effect on the enzyme activity, which results in a strong reduction of the carboxylation reaction rate. Due to this, this strategy is unfeasible, and not even the equilibrium yield is reached without adding enzymes in excess. Attempting to enhance conversion via the addition of TBA-Br for in situ product precipitation reduced the conversion at elevated pressure to values below the ones obtained when not using the quaternary ammonium salt. It is expected that CO_2_ carbamylation of amino acids on the enzyme backbone changes its conformation, reducing its activity. When adding TBA-Br, the formed carbamates could form electrostatic interactions with it, reducing the activity further. The underlying mechanism of inhibition needs further investigation. It is concluded that the application of elevated pressure on the enzymatic carboxylation of resorcinol is not an expedient solution at this stage.

## Data Availability

Data are contained within the article and raw data is available on request from the corresponding author.

## Abbreviations

2,6-DHBD, 2,6-dihydroxybenzoic acid decarboxylase from *Rhizobium* sp.; 2,6-DHBA, 2,6-dihydroxybenzoic acid; TEA, triethanolamine; DIC, dissolved inorganic carbon concentration; TBA-Br, tetrabutylammonium bromide; ‘CO_2_’, CO_2_ and H_2_CO_3_.
